# Quality Characteristics of Sustainable High-Performance Concrete Formulated from Binary, Ternary, and Quaternary Supplementary Cementitious Materials Under Various Curing Conditions

**DOI:** 10.3390/ma17235831

**Published:** 2024-11-27

**Authors:** Mohammad Iqbal Khan, Yassir M. Abbas, Galal Fares

**Affiliations:** Department of Civil Engineering, College of Engineering, King Saud University, P.O. Box 800, Riyadh 11421, Saudi Arabia; miqbal@ksu.edu.sa (M.I.K.);

**Keywords:** binary, ternary and quaternary systems, ultrafine, fillers, filling effect, pozzolanic activity

## Abstract

The formulation of binary, ternary, and quaternary supplementary cementitious materials (SCMs) on an optimized silica fume amount using fly ash, ultrafine (MQ), and limestone powders (LS) is the most sustainable approach to recycling these types of solid wastes for durable concrete. The optimum replacement level of 10% silica fume was blended with different replacement levels of 5, 8, 10, and 15% MQ to formulate different ternary mixes to evaluate the filling effect of MQ. Different ternary mixes containing 10% silica fume and 5, 10, and 15% LS were also produced to examine the effectiveness of both ternary mixtures with either MQ or LS. The quaternary mixtures with 10% silica fume optimized with 20% fly ash and 10% MQ or 10% LS were evaluated for compressive strength, chloride permeability, and porosity. The MQ showed the best filling effect compared to LS. The hot curing conditions significantly enhanced the performance of ternary and quaternary mixtures. Two effects of fillers were observed: the diluting effect brought on by replacement levels and the enhanced filling effect. At early curing, the strength loss resulting from the high replacement level was around 39%; however, this drop could be minimized to approximately 7% under hot curing conditions. It has been demonstrated that the binary, ternary, and quaternary systems offer the best solution to the environmental and durability issues caused by cement. The economic analysis highlights that optimized HPC mixtures with SCMs and fillers, particularly the quaternary mix, achieve superior cost-efficiency and mechanical performance, demonstrating their potential for sustainable and high-performance engineering applications.

## 1. Introduction

Concrete is the most widely used construction material globally, with annual consumption exceeding 30 billion tons, making it second only to water [1,2]. This immense demand is driven by rapid population growth, urbanization, industrialization, and the development of megacities. Ordinary Portland cement (PC), the primary binder in concrete, has seen an exponential increase in production, rising from 10 million tons in 1900 to approximately 3.5 billion tons by 2016, with China producing 2.4 billion tons annually, followed by India and the United States [3,4,5,6]. The global consumption of cement currently stands at approximately 4.2 billion metric tons annually [7]. With the accelerating pace of urbanization, population growth, and infrastructure development, this demand is projected to surpass nearly 6 billion metric tons annually by 2050 [8]. However, the extensive use of OPC has significant environmental implications, including high energy consumption and greenhouse gas emissions, necessitating sustainable alternatives.

To meet the challenges of modern construction while minimizing environmental impacts, high-performance concrete (HPC) has gained prominence. HPC is characterized by superior strength, workability, and durability under various conditions, making it suitable for critical infrastructure and extreme environments [9,10,11]. Its performance is primarily attributed to the inclusion of supplementary cementitious materials (SCMs) such as silica fume (SF), fly ash (FA), and granulated blast furnace slag, which enhance mechanical and durability properties by improving the microstructure of the cementitious matrix [4,10,11,12]. The addition of ultrafine fillers, such as micro-quartz (MQ), further contributes to particle packing, water demand reduction, and interfacial transition zone refinement, ultimately enhancing strength and durability [13,14].

SCMs offer several benefits beyond reducing the reliance on OPC. For instance, SF significantly enhances compressive strength through pozzolanic reactions, which consume portlandite to form secondary calcium silicate hydrate gel [15]. FA, rich in amorphous aluminosilicates, reduces the heat of hydration, improves workability, and enhances long-term durability by filling voids and contributing to pozzolanic activity [16,17,18]. The combined use of SF and FA in ternary blends has been shown to optimize both mechanical and durability properties, achieving high compressive strength and reduced chloride permeability [18,19,20]. Ternary mixtures, such as those incorporating 9% SF and 26% FA, have significantly improved impermeability, with chloride ion penetration charges as low as 500 coulombs [21,22].

MQ and LS are non-reactive but positively influence HPC properties by increasing packing density and reducing interstitial voids [23]. Their fine particle size enhances the interfacial bond between the cement matrix and aggregates, improving mechanical performance [24]. These fillers also act as nucleation sites, accelerating the hydration reaction and promoting early-age strength development [25,26]. However, their effectiveness diminishes beyond certain replacement levels due to reduced cementitious reactivity, as observed in mixes with excessive filler content [27].

Despite the benefits, the synergistic effects of SCMs and MQ on HPC properties remain only partially understood, particularly regarding durability-related parameters under varying environmental conditions [27,28]. This gap is critical for achieving sustainable development goals in the construction sector, as SCM-based HPC can address both environmental and economic challenges. For instance, ternary and quaternary blends of SCMs have been shown to mitigate alkali–silica reactions and chloride intrusion, key factors in enhancing the service life of concrete structures [29,30,31,32,33].

It is worth mentioning the influence of SCM mixes on the thermal expansion and contraction of concrete. For this reason, it is important to assess the thermal expansion of concrete, as it can lead to joint blocking, while its contraction can lead to cracking. Different methods are used in the thermal conductivity measurements of concrete adopted by ASTM, such as ASTM C518 and D5334. However, it is well reported that there is a significant difference between the results obtained from both methods [34]. It is accordingly recommended to use one for consistent and accurate comparison. Both silicate phases, C3S and C2S, have a similar thermal conductivity of 3.35 and 3.45 W/m·K, respectively [35]. The effect of increasing the slag content from 36% to 50% is reported to cause an increase in the thermal conductivity from 0.648 to 1.023 W/m·K, while the increase in the fly ash content from 26% to 35% is reported to lead to 0.534 to 0.689 W/m·K [34]. Moreover, the presence of FA, SF, and slag improves high-temperature resistance [36].

A literature review conducted earlier revealed that little literature exists on using MQ in concrete to enhance its durability-related properties. To advance the understanding of HPC’s performance, the current study examined the benefits gained from the use of various SCMs in HPC in binary, ternary, and quaternary combinations. We used four different SCMs: SF, MQ, LS, and FA. In this study, we examined the compressive strength, chloride permeability, and porosity of 12 SCM-based HPCs in comparison to a comparable reference. The results aim to provide insights into optimizing HPC mixtures for sustainable and high-performance applications, contributing to reduced environmental impacts and improved infrastructure longevity.

## 2. Materials

**Figure 1:**
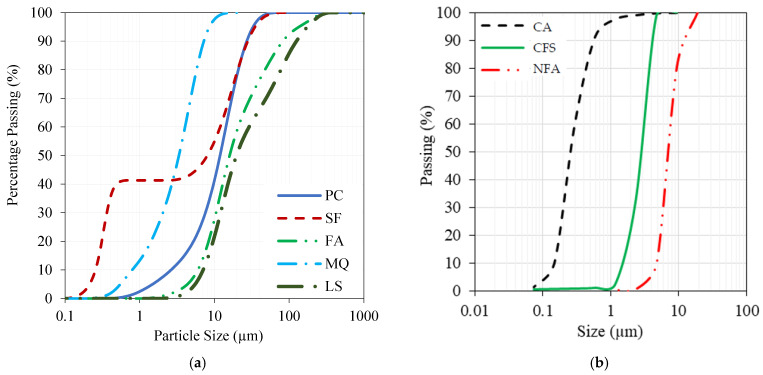
The laser particle size distribution of (**a**) fine powders and sieve analysis of (**b**) aggregates [38].

PC satisfying the ASTM C150 [37] specifications was blended with SF and FA as supplementary cementitious materials in addition to MQ and limestone powder (LS). The particle-size distribution of PC, SF, FA, MQ, and LS revealed that they had median grain sizes of approximately 13, 8, 15, 3.5, and 19 µm, respectively. The chemical and physical properties of the powders used in this study are presented in Table 1. The laser particle-size distribution analysis of the same powders is shown in Figure 1a, while the sieve analysis of aggregates used is shown in Figure 1b.

**Figure 2:**
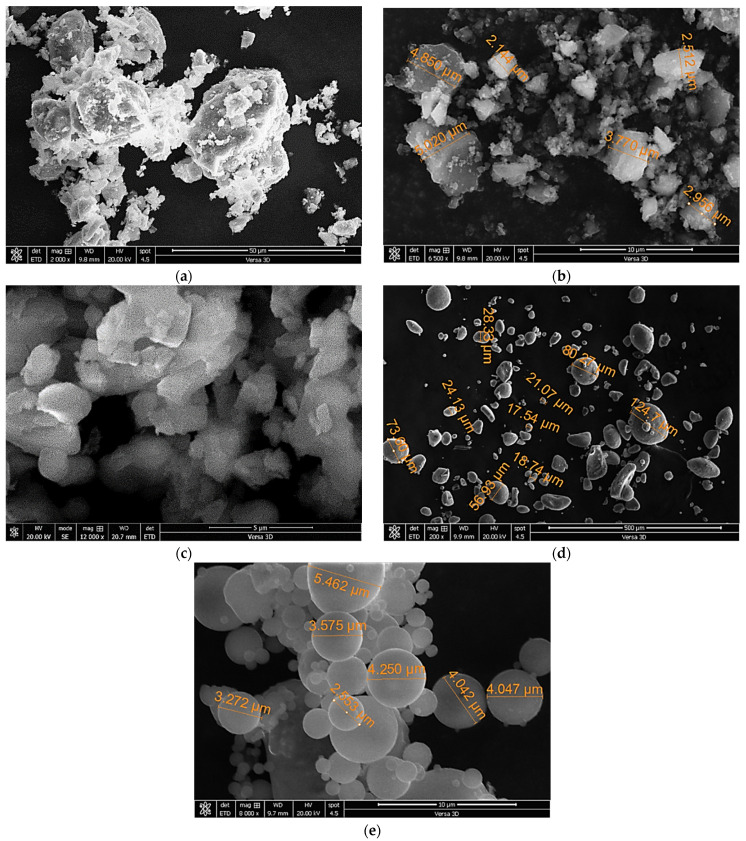
Photomicrographs of (**a**) PC, (**b**) MQ, (**c**) PC, (**d**) FA, and (**e**) SF.

Microstructural analysis of the cementitious and filler powders using scanning electron microscopy is depicted in Figure 2. The angularity of the powder due to the grinding process of cement, quartz, and LS raw materials is evident, as shown in Figure 2a, Figure 2b, and Figure 2c, respectively. Cement particles have different sizes, with an average elongated size of about 30 µm due to their increased hardness, while the MQ particles with average sizes of around 2 to 5 µm are evident, as shown in Figure 2b. Condensed silica fume particles are noted in Figure 2d, while the cenosphere particles of FA are shown in Figure 2e.

The workability of concrete mixtures was adjusted using a polycarboxylic ether-based superplasticizer (SP) with a relative density of 1.1 and a solid content of 36%. The SP dosage was calculated as a percentage of the binder content. To achieve the desired grain distribution of fine aggregates with an acceptable fine modulus, a mixture of natural fine sand (NFAs) and crushed fine aggregates (CFAs) in a proportion of 65% and 35%, respectively, was used. The fine modulus, calculated as 2.54, was obtained by performing a sieve analysis on the combined fractions of NFA and CFA, following ASTM C 136 [39]. This involved summing the percentages of material retained on standard sieves and dividing the total by 100. The maximum aggregate size for the coarse aggregate (CA) was below 10 mm. NFA, CFA, and CA densities were 2.63, 2.68, and 2.65, respectively. The sieve analysis of the aggregates is previously presented in Figure 1b.

## 3. Methodology

### 3.1. Preparation Scheme

**Figure 3:**
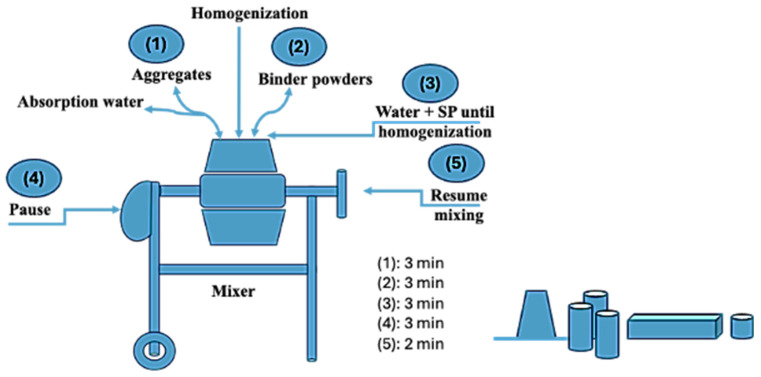
Schematic representation of mixing procedure and sample preparation.

At the beginning of concrete mixture preparation, all aggregates were homogenized in the mixer drum for 3 min, where absorption water was added at the last minute. This step was followed by adding the fine powders composing the cementitious binder and mixing until apparent homogenization was obtained, which usually takes between 2 and 3 min. Both mixing water and SP were added to the dry mix while the mixer was on for three minutes, followed by a three-minute pause followed by two-minute mixing to reach the final stage and stopping the mixer to measure the slump and cast different specimens for curing and testing. The normal curing conditions (N) of 23 °C and relative humidity of 100% were applied during the specimens’ curing. At the same time, hot curing conditions of 40 °C and low relative humidity below 20% were used for hot curing conditions (H). Specimen molds for compressive strength and chloride permeability were prepared as per ASTM C39 [40] and ASTM C1202 [41], respectively. ASTM C1437 [42] was used to measure the slump of concrete. The slump of all mixes needed to be between 170 and 225mm throughout the mix batch process to ensure that the concrete remained in working order. The temperatures and unit weights of all the samples were measured using ASTM C1064 [43] and ASTM C138 [44]. Illustrated in Figure 3 is a detailed schematic outlining the process for mixing and the subsequent preparation of samples.

### 3.2. Detail of Mixtures

A total of 13 mixtures were tested to determine the most effective dosage of SF, FA, MQ, and LS (Table 2). The table provides a detailed classification of concrete mixtures based on their specific compositions, as indicated by the mix IDs. Here, the designation CTRL represents the control mix, which excludes any SCMs or MQ. In this study, SF10 refers to a mixture containing SF at a replacement level of 10% (vol.), while SF10-FA20 denotes a composition incorporating 10% SF combined with 20% FA. These specific replacement levels were selected based on prior investigations by the authors, which identified 10% SF and 20% FA as optimal levels for improving the mechanical performance and sustainability of the mixtures. Further details supporting this optimization can be found in [45]. Additionally, compositions labeled as “SF10-MQx” or “SF10-LSy” feature 10% SF combined with x% MQ or y% LS, respectively. Notably, the LS percentages were chosen to utilize their dual role as fillers and reactive materials forming carboaluminates, which enhance mechanical properties. The exclusion of 8% LS was a deliberate choice to focus on replacement levels that displayed clearer performance trends in line with this study’s scope. Moreover, mixtures such as SF10-FA20-MQx or SF10-FA20-LSy represent ternary blends containing SCMs and MQ or LS, respectively. The mixes were cured under two different environmental conditions in the present study. Throughout all the mixtures shown in Table 2, the ratio of water to cementitious material was kept constant at 0.3. In addition, the coarse aggregate content was maintained constant at 1056 kg per cubic meter for all the mixtures kg/m^3^. Table 2 shows the slump, temperature, and unit weight of the concrete mixtures in their fresh state. Their ranges were 185–206 mm, 19.2–25.2 °C, and 2394–2441 kg/m^3^, respectively.

**Figure 4:**
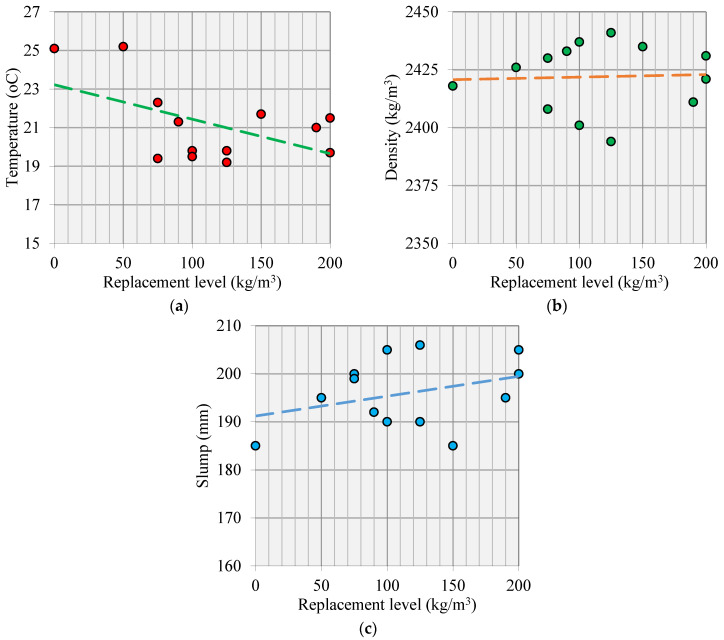
Relationship between replacement level of SCMs and (**a**) fresh concrete temperature, (**b**) fresh concrete density, and (**c**) slump of fresh concrete.

A discernible linear correlation emerges between the replacement levels of supplementary cementitious materials (SCMs) and the temperatures of fresh concrete. In contrast, the density of fresh concrete exhibits consistent fluctuations around a mean value, as illustrated in Figure 4. The replacement level significantly influences the cement content, subsequently impacting the amount released through cement hydration. Unlike temperature, the variation in density is not strictly tied to the fluctuation in SCM replacement levels. This divergence is attributed to variations in density and compensatory effects by aggregates. The impact of the replacement level on the slump reveals a discernible increasing trend on average, as depicted in Figure 4c.

### 3.3. Testing Scheme

**Figure 5:**
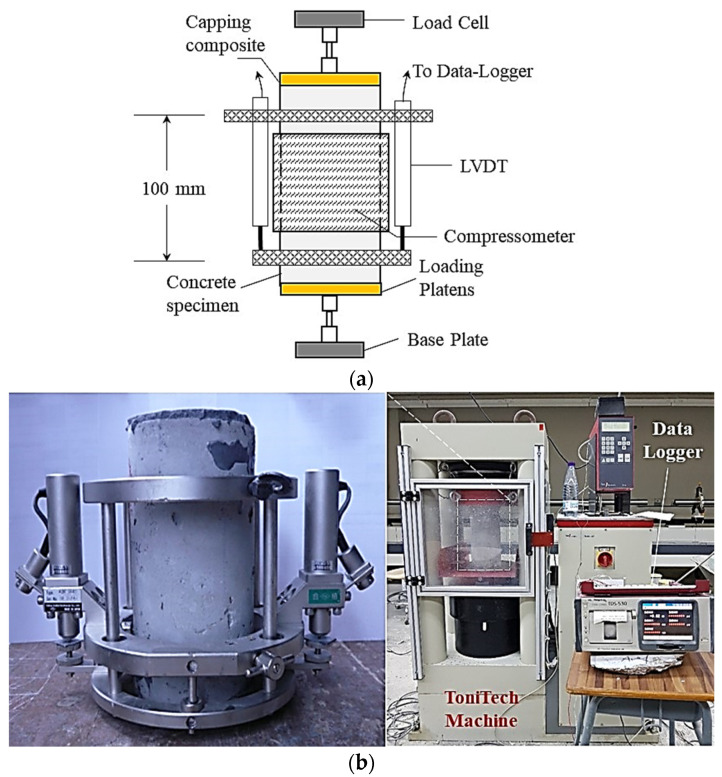
Compressive strength and MOE measurements: (**a**) graphical representation and (**b**) actual setup.

Sulfur capping was employed to ensure equal loading distribution during the preparation of concrete cylinders for compressive strength testing. The specimens’ compressive strength and modulus of elasticity (MOE) were evaluated at 28 days following ASTM C39 [40,46] and ASTM C469 [44], respectively, using a ToniTech (Tonitech Equipment and Chemical Co., Ltd., Bangkok, Thailand) compression testing machine with a 3000 kN capacity. The displacement-controlled testing was conducted at a rate of 2.5 × 10^−3^ mm/s, which corresponds to a stress rate of approximately 0.6 N/mm^2^/s, consistent with the preferred range of (0.6 ± 0.2) MPa/s recommended by the British Standard (BS 1881 [47]) for MOE testing. This approach is further supported by Akiije [48]. Axial strain measurements were obtained using two linear variable displacement transducers (LVDTs), while in-plane and transverse strains were estimated using compressometer rings positioned 50 mm from both sides of the specimen’s center, as illustrated in Figure 5. The mean results of three specimens are reported in this study.

**Figure 6:**
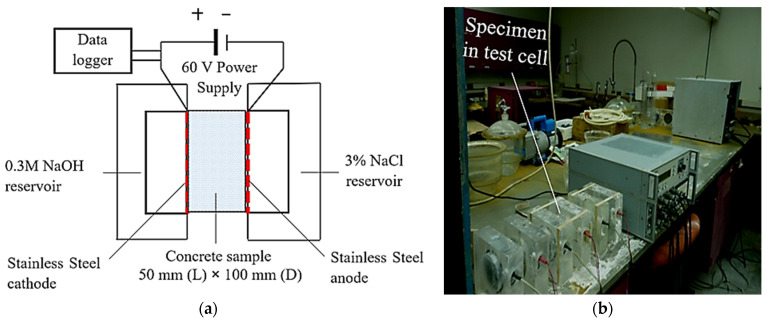
RCPT setup: (**a**) graphical representation and (**b**) actual test setup.

The rapid chloride permeability test (RCPT) was conducted as detailed in ASTM C 1202 [41] and discussed by Zagar [49]. This test has been criticized for not accurately estimating the diffusion properties [50]; however, it can be used for parametric comparison. The test setup and testing conditions are depicted in Figure 6. Each chloride permeability value represents an average of three specimens.

The RILEM CPC 11.3 method was followed to estimate the porosity of concrete. Specimens of similar dimensions to those used in RCPT as per ASTM C1202 [41] were used in this test. The porosity was estimated based on the ratio of the differences in masses of the specimens under various humidity conditions. The ratio of the water-saturated specimen’s mass minus the oven-dry specimen’s mass to the water-saturated specimen’s mass minus the water-suspended specimen’s mass is defined as the specimen’s porosity. For each type of mixture, porosity measurements (Concrete Testing Solutions. Industrial Area Phase 2, Riyadh, Saudi Arabia) were conducted on three specimens, and the results presented in the manuscript represent the average values obtained from these tests.

## 4. Results and Discussion

### 4.1. Compression Test Results

#### 4.1.1. Compressive Strength of SF-MQ Ternary Concrete Mixtures

**Figure 7:**
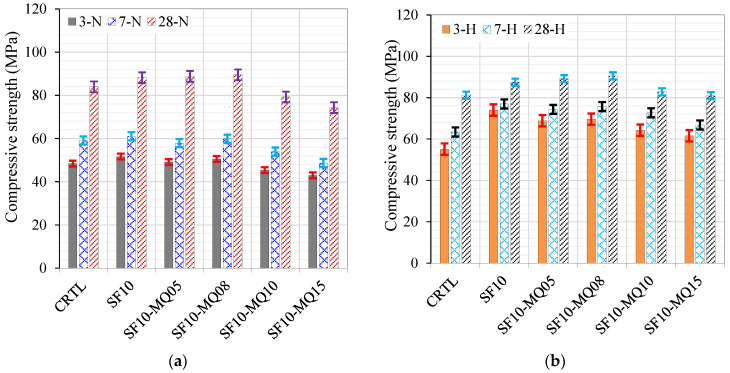
Compressive strength as a function of replacement level under two curing conditions: (**a**) normal conditions (N) and (**b**) hot curing conditions (H).

To evaluate the effect of MQ in the absence and presence of SF, the control and the binary with 10% mixes were used for comparison. The effect of adding four dosages of MQ (5, 8, 10, and 15% QM) in the presence of 10% SF was investigated under normal curing conditions and curing ages of 3, 7, and 28 days, as demonstrated in Figure 7a. The enhancing effect of 10% SF is remarkable, which slightly increased gradually until the replacement level of MQ was 8%, followed by a gradual reduction of 5.5% and 11% with additional replacement levels of 10 and 15%, respectively, compared to the control mix.

**Figure 8:**
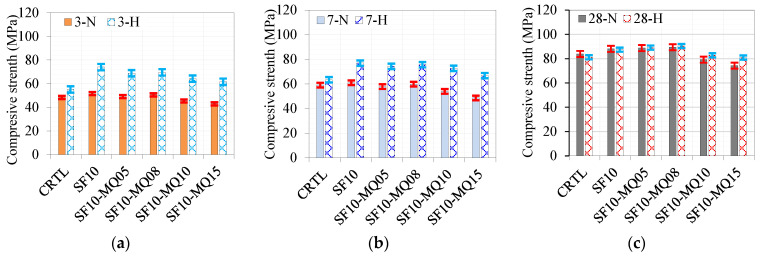
Effect of binder composition on strength at different curing ages of (**a**) 3 days, (**b**) 7 days, and (**c**) 28 days.

A similar trend was observed under the hot curing conditions except for the ternary mixes with 10% and 15% QM. The 10% MQ mix showed higher strength than the control, while the mix with 15% QM gave strength comparable to the control mix due to the accelerating effect of the hot curing conditions. A comparison of the development of the compressive strength at 3, 7, and 28 days under normal and hot curing conditions is shown in Figure 8a, Figure 8b, and Figure 8c, respectively. The effect of hot curing is remarkable at curing ages of 3 and 7 days compared to the control and normally cured mixes, as depicted in Figure 8a,b, respectively. However, there is no difference between the strength values at a curing age of 28 days. The optimal filling effect was noted at a replacement level of 8% QM, where the enhancement in strength reached 26%, as demonstrated in Figure 8c. Further replacement levels of 10% and 15% QM lead to a notable reduction in strength due to the dilution effect.

#### 4.1.2. Compressive Strength of SF-MQ-FA Quaternary Concrete Mixtures

**Figure 9:**
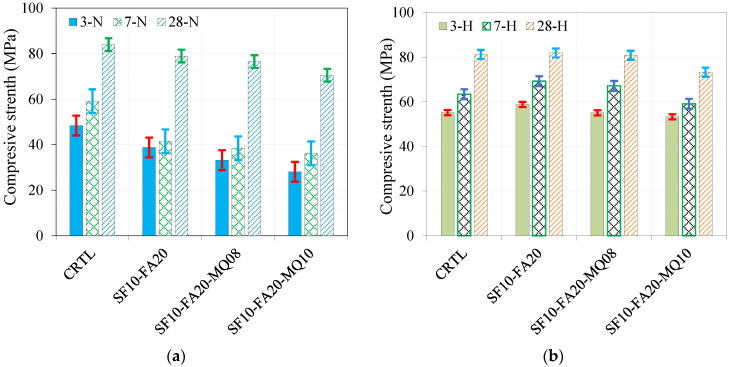
Development of compressive strength with time under (**a**) normal curing conditions and (**b**) hot curing conditions.

The formulation of the ternary mix of 10% SF and 20% FA yielded a lower rate of early strength development. The incorporation of 8% and 10% MQ to formulate the quaternary system leads to a gradual reduction of up to 17% in strength under normal curing conditions, as presented in Figure 9a. The effect of hot curing conditions was substantial and led to a significant modification in the trend of the ternary system with 10% SF and 20% FA as a result of the accelerating effect of the heat input with comparable results to the quaternary mix with 10% SF, 20% FA, and 8% MQ, as depicted in Figure 9b. Even the quaternary system with 10% SF, 20% FA, and 10% MQ showed an improved strength of 4% when compared to the corresponding mix under normal curing conditions, as inferred from Figure 9a,b.

**Figure 10:**
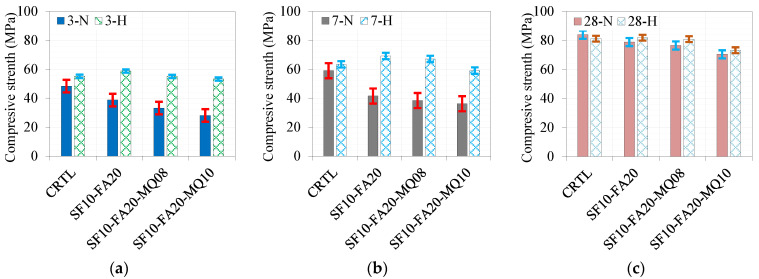
Comparison of compressive strength of different ternary and quaternary mixes under various curing conditions at (**a**) 3 days, (**b**) 7 days, and (**c**) 28 days.

A comparison of the effect of curing conditions at different curing ages of 3, 7, and 28 days is given in Figure 10a, Figure 10b, and Figure 10c, respectively. The enhancing effect of the curing conditions is prevalent at 3 and 7 days, as demonstrated in Figure 7b and Figure 10a, respectively. Under normal curing conditions, the reduction in strength reached a maximum value of approximately 39% at 7 days. In contrast, this reduction was significantly minimized to about 7% under hot curing conditions due to the accelerated hydration process. Hot curing promotes faster hydration by expediting the reaction between water and the binder, particularly during early-age curing in concrete with high binder content. In such systems, elevated temperatures enable the rapid formation of hydration products, enhancing early strength development. However, at 28 days, this advantage is diminished due to the combined effect of the limited relative humidity under hot curing conditions, which hinders sustained hydration and the natural progression of strength development under normal curing. This interplay results in comparable strengths for both normal and hot-cured samples, as illustrated in Figure 10c. Additionally, in the Gulf region’s climatic conditions, hot curing is especially relevant to mitigate excessive heat accumulation in mass concrete. Incorporating fillers and supplementary cementitious materials effectively reduces cement content and prevents potential cracking and durability issues associated with high heat release.

#### 4.1.3. Development of Compressive Strength of Ternary and Quaternary Concrete Mixtures

**Figure 11:**
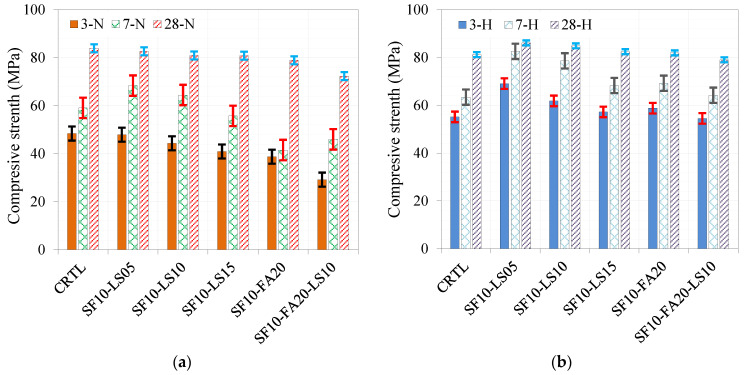
Development of compressive strength over time under different curing conditions of (**a**) normal curing conditions and (**b**) hot curing conditions.

The incorporation of LS and FA to formulate ternary mixtures of SF and LS and quaternary mixtures of SF, LS, and FA was conducted and investigated for an optimum mixture, as shown in Figure 11. Compared to the control mixture under normal curing conditions, the ternary mixtures of SF with 5%, 10%, and 15% LS show a limited reduction to a maximum of about 6%. However, the quaternary system was formulated using a 10% SF, 20% FA, and 10% LS mixture, which led to a reduction of 14% in compressive strength, as demonstrated in Figure 11a. On the other hand, the effect of hot curing conditions on the ternary mixture of 10% SF and 5% LS led to an enhancement of 6%, while enhancements of 4.5%, 1.5%, and 1% were noted for the ternary mixtures of 10% SF and 10% LS, 10% SF and 15% LS, and 10% SF and 20% FA, respectively, as depicted in Figure 11b. The quaternary mixture of 10% SF, 20% FA, and 10% LS showed a marginal reduction of 2.6% compared to the control mixture. To further contextualize these findings, previous research [51] has demonstrated that fine fillers, such as LS and red sandstone powders, can synergistically enhance hydration and the formation of carbon aluminates in later stages, leading to improved microstructure and interface transition zones. The study also highlighted that the combined use of such fillers compensates for dilution effects, enhancing compressive strength.

**Figure 12:**
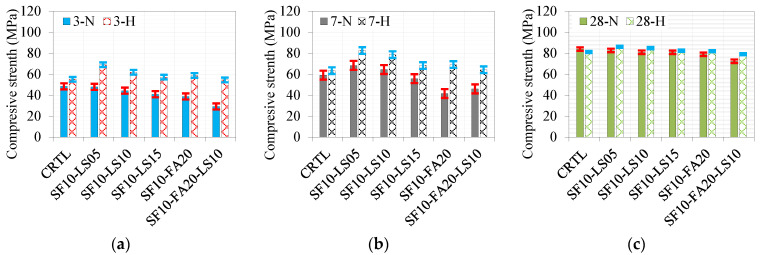
Comparison of compressive strength of different ternary and quaternary mixes under different curing conditions at (**a**) 3 days, (**b**) 7 days, and (**c**) 28 days.

A comparison of the effect of curing conditions at different curing ages of 3, 7, and 28 days is given in Figure 12a, Figure 12b, and Figure 12c, respectively. At curing ages of 3 and 7 days, the effect of hot curing conditions outperformed the normal curing conditions, as depicted in Figure 10b and Figure 12a, respectively. The difference reduces substantially at 28 days, as demonstrated in Figure 12c. The improving effect of hot curing conditions is notable for the quaternary mixture of 10% SF, 20% FA, and 10% LS at all ages.

#### 4.1.4. Compressive Strength Variation with Time

**Figure 13:**
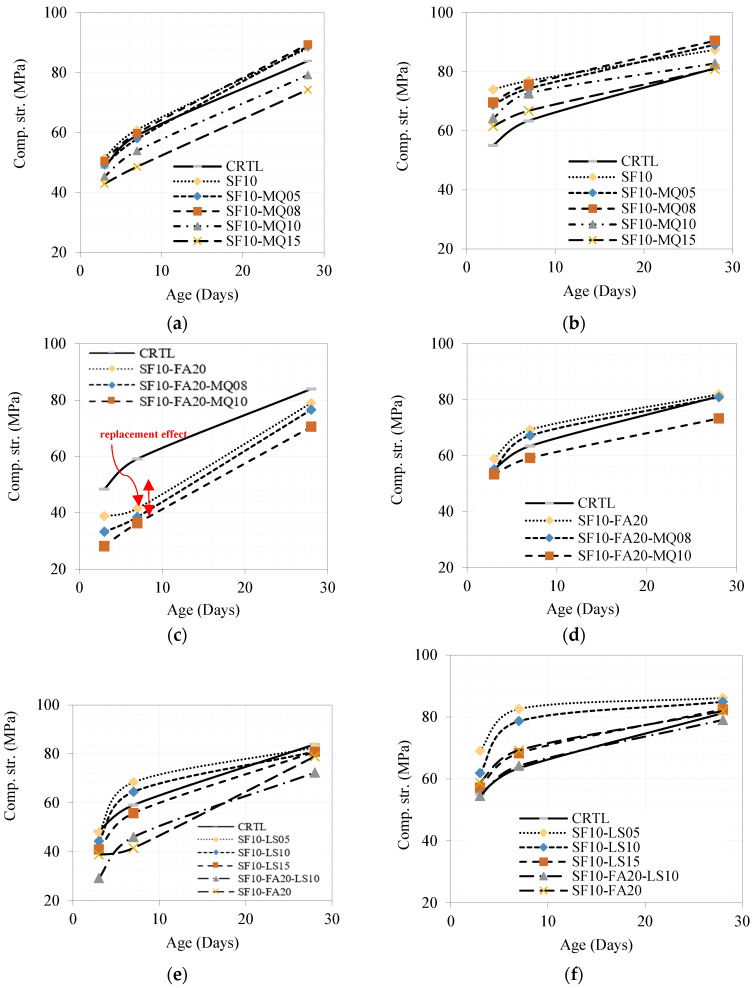
Development of compressive strength over time of different ternary and quaternary mixtures under normal and hot curing conditions. (**a**) Normal curing; (**b**) Hot curing; (**c**) Normal curing; (**d**) Hot curing; (**e**) Normal curing; (**f**) Hot curing.

The development of compressive strength of the ternary mixtures of 10% SF with different replacement levels of 5, 8, 10, and 15% MQ compared to the binary mixture with 10% SF and the control one under normal and hot conditions is shown in Figure 13a,b, respectively. The enhancing effect of curing conditions is revealed in this comparison. From the comparison, the ternary mixture of 10% SF and 8% MQ is the optimum mixture whose strength is much improved under the hot curing conditions, as shown in Figure 13b. Similarly, a comparison between different quaternary mixtures of 10% SF, 20% FA, and 8% MQ and 10% SF, 20% FA, and 10% MQ with replacement levels exceeding 38% compared to the ternary mixture with 10% SF and 20% FA as well as the control one was conducted as shown in Figure 13c,d under normal and hot curing conditions, respectively. The effect of replacement level, especially at early ages, is notable under normal curing conditions, as demonstrated in Figure 13c. The supportive effect of hot curing conditions is shown in Figure 13d. The ternary and quaternary mixtures with LS under normal and hot curing conditions are given in Figure 13e,f, respectively. The comparison was conducted between the control, the ternary mixtures of 10% SF and 5%, 10%, and 15% LS concerning the ternary mixture of 10% SF and 20% FA as well as the quaternary one with 10% SF, 20% FA, and 10% LS. The ternary mixtures of 10% SF, 5% LS, and 10% LS were the best performing, with 10% SF and 5% LS being the optimum.

### 4.2. Rapid Chloride-Ion Permeability Test Results

**Figure 14:**
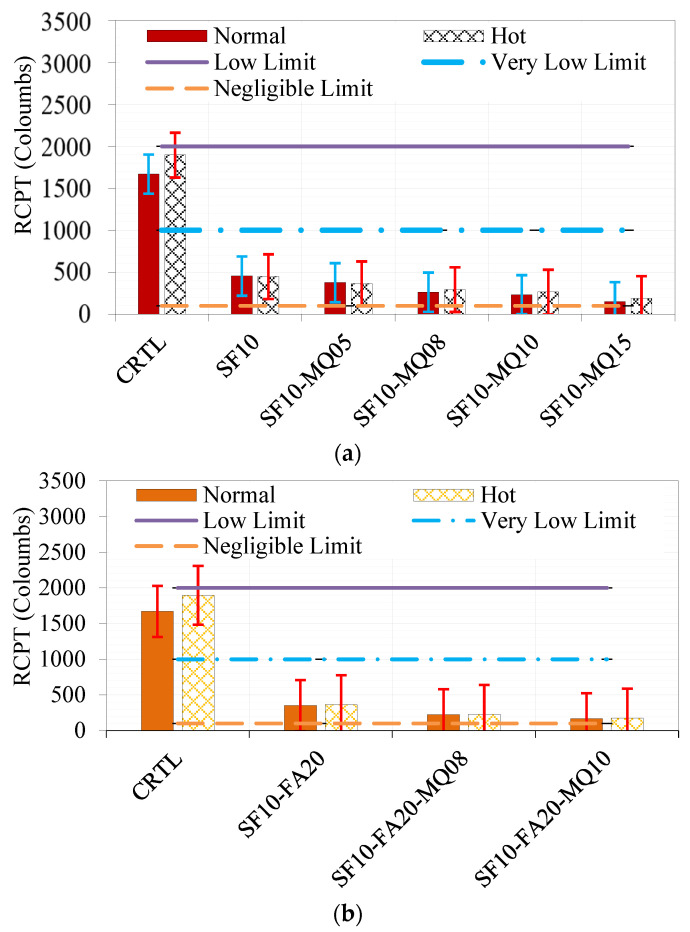

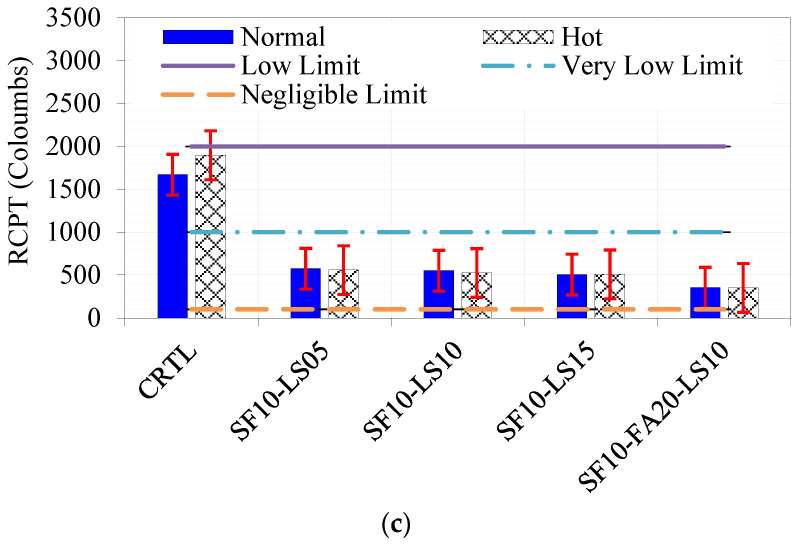
Rapid chloride permeability test (RCPT) of different binary, ternary, and quaternary mixtures: (**a**) SF-MQ-based mixtures, (**b**) SF-FA-MQ mixtures, and (**c**) SF-FA-LS mixtures under normal and hot curing conditions.

The rapid chloride permeability test (ASTM C1202 [41]) measures the electric charge passed through concrete in coulombs, a key indicator of its permeability and durability. Concrete with higher permeability allows for harmful chemicals, such as chlorides and sulfates, to penetrate more easily, causing steel corrosion and reducing structural integrity. Figure 14a shows that the control mixture exhibited a charge exceeding 1500 coulombs, indicating high permeability. In contrast, binary mixtures with 10% SF and ternary mixtures with 10% SF and 5, 8, 10, or 15% MQ exhibited charges below 500 coulombs, decreasing proportionally with the replacement level of MQ. Similarly, Figure 14b shows that the control and binary mixture with 10% SF and 20% FA had significantly higher permeability than quaternary mixtures incorporating 10% SF, 20% FA, and 8 or 10% MQ, where charges fell below 200 coulombs. Figure 14c highlights the differences in efficiency between MQ and LS due to their particle size, texture, and chemical composition. While MQ mixtures demonstrated lower permeability, with charges consistently below 500 coulombs, LS mixtures showed slightly higher values, although quaternary mixtures containing 10% SF, 20% FA, and 10% LS reduced the charge to below 400 coulombs. These results demonstrate the superior filling effect and durability enhancement provided by MQ compared to LS.

### 4.3. Porosity Test Results

**Figure 15:**
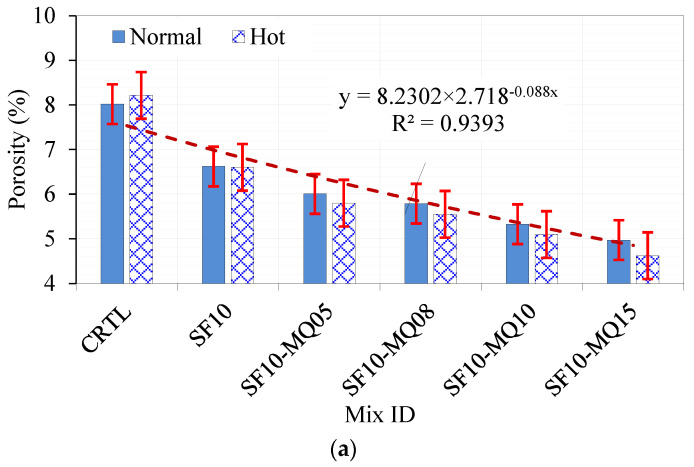

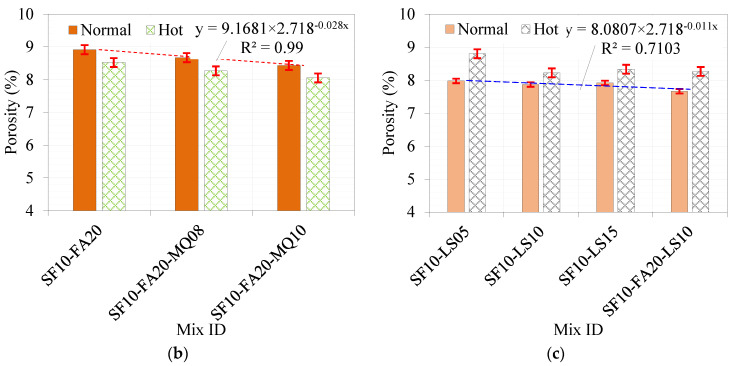
Results of porosity test for (**a**) SF-MQ ternary mixtures, (**b**) SF-FA-MQ quaternary mixtures, and (**c**) SF-FA-LS ternary and quaternary mixtures under both normal and hot curing conditions (Note: ‘x’ represents the corresponding mix number in each figure (e.g., x=2 for SF10 mix in Figure 14a)).

An exponential relationship is found in the group of mixtures of the control, binary of 10% SF, and ternary mixtures of 10% SF with different replacement levels of 5, 8, 10, and 15% MQ referring to a leveling-off effect with a rapid reduction in porosity with replacement level and a coefficient of variation R^2^ of 0.94. This relationship means that after a certain level of replacement, any further replacement will not affect the porosity anymore. The porosity range observed in these mixtures varied from 4.6% to 8.9%. A similar trend is also noted in the ternary mixture with 10% SF and 20% FA, the quaternary mixtures with 10 and 20% FA, and different replacement levels of 8% and 10% MQ. A similar exponential trend is observed in this series of mixtures. It is worth noting that the hot curing conditions led to a slight increase in the porosity of the control mixture due to the accelerated hydration, leading to increased porosity. It was also noticed that the porosity of all MQ-based mixtures cured under hot conditions showed lower porosity values than the mixtures cured under normal curing conditions, as demonstrated in Figure 15a,b. However, the porosity stayed near 8% due to the high replacement level. Notably, the porosity increased with the addition of LS under hot curing conditions, which was attributed to both the accelerated hydration and replacement level, as was the case with the control mixture, as inferred from Figure 15a and shown in Figure 15c.

### 4.4. Permeability and Porosity Relations

**Figure 16:**
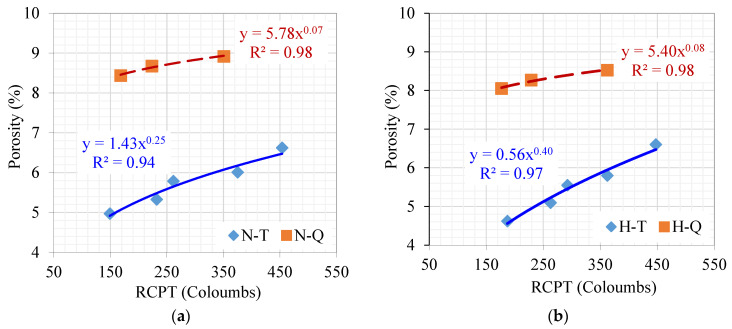
A relationship between porosity as a function of rapid chloride permeability values and under (**a**) normal and (**b**) hot curing conditions.

The relationships between porosity as a function of porosity for the ternary (T) and quaternary (Q) mixtures under normal (N) and hot (H) curing conditions were established, as presented in Figure 16a,b, respectively. The effect of increased replacement level on porosity as a function of permeability, regardless of the curing conditions, is demonstrated in Figure 16a,b, where the maximum porosity reaches nearly 9%. The ternary mixtures under normal or hot curing conditions showed lower porosity compared to the quaternary mixtures under normal and hot curing conditions. Regardless of the curing conditions, the replacement level has a significant effect on both the permeability and porosity. The porosity here can be a reflection of the need for water to resume hydration and is not just related to the real porosity of the system. All the relationships are shown to be power functions that also refer to a leveling-off effect to reach the maximum value, after which no significant increase is noted.

## 5. Economic Feasibility Investigation

In this study, the economic feasibility of incorporating SCMs and fillers in HPC was assessed through a cost–performance analysis of various concrete mixtures. This study focused on determining the cost-effectiveness of different mix designs while maintaining or enhancing mechanical performance, represented by compressive strength under both normal and hot curing conditions. This analysis enables a better understanding of the practical application potential of these mixtures in engineering projects. The analysis involved calculating the material costs for each mixture based on compiled market prices and evaluating their performance through cost-normalized indices. These indices, derived by normalizing the compressive strength against the cost of each mixture, provide an integrated measure of cost-efficiency. Here, compressive strength was prioritized as the primary performance metric because it is the most critical indicator of structural performance, directly correlating with cost-efficiency and universally used in design standards. While durability properties were assessed, strength serves as a gateway metric, ensuring practical relevance and aligning with industry priorities for material selection and application. The costs of various concrete-making materials were compiled from multiple sources as summarized in Table 3.

**Figure 17:**
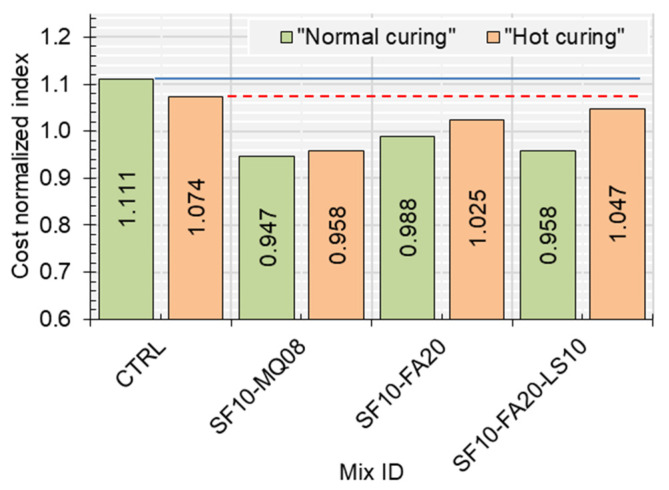
Cost-efficiency metrics for the optimized concrete mixtures.

The cost-normalized index for each mixture was computed using the formula $\left[Costnormalizedindex(MPa.m^{3}/€)=Compressivestrength(MPa)/Cost(€/m^{3})\right]$. The details of this analysis are presented in Table 4 and Figure 17. The CTRL, with a cost of EUR 75.6/m³, attained cost-normalized indices of 1.111 and 1.074 for normal and hot curing, respectively. The optimized binary mix (SF10-FA20), with a slightly higher cost of EUR 79.9/m³, achieved indices of 0.988 and 1.025, demonstrating its competitive cost-efficiency while maintaining superior performance under hot curing conditions. Similarly, the ternary mix (SF10-MQ08), despite a higher cost of EUR 94.5/m³, achieved indices of 0.947 and 0.958, reflecting its ability to provide enhanced strength in demanding applications, particularly under hot curing.

The optimum quaternary mix (SF10-FA20-LS10) emerged as a standout option, achieving cost-normalized indices of 0.958 and 1.047 under normal and hot curing conditions, respectively, at the same cost as the control mix (EUR 75.6/m³). This demonstrates its remarkable balance of cost and performance, especially in environments requiring durability under elevated temperatures. When compared to the control mix, all optimized combinations showed tailored improvements in specific conditions, which reflects the synergistic effects of SCMs and fillers on performance metrics. This analysis highlights the rational trade-offs between cost and performance of the optimized mixtures. While the control mix remains economically efficient, the optimized combinations offer targeted enhancements in mechanical properties and cost-efficiency, depending on the curing environment and project requirements.

## 6. Conclusions, Limitations, and Future Studies

From this study, the importance of the filler, either regular microsize or MQ, is highlighted. The effect of different curing conditions on concrete properties is shown. The following can be concluded:

(1) The fresh properties are affected by the replacement levels of the SCMs. The temperature of the mixes decreases with an increase in the replacement level. The slump increases with an increase in the replacement level of SCMs. The effect on the concrete density is affected by many parameters, including the type, nature, and volume of SCM, in addition to the fine and coarse aggregate content.
(2) Strength development is substantially affected by the replacement level of SCMs. The presence of MQ enhanced the strength until a replacement level of 8%. The incorporation of LS into compressive strength is noted to be slightly accompanied by a gradual reduction in strength that could be compensated using hot curing conditions. The quaternary cementitious systems led to a substantial reduction in strength that could be overcome using hot curing conditions.
(3) The enhancing effect of SCMs on reducing chloride permeability is noted in all binary, ternary, and quaternary mixtures compared to the control mixture.
(4) Porosity based on water absorption decreases with binary and ternary systems until the optimum replacement levels are reached, while it increases with quaternary systems due to reduced hydration reactions. Similarly, the effect of hot curing conditions increases water absorption due to the early accelerated hydration, which leads to an increased pore system.
(5) The mixtures investigated demonstrated significantly improved impermeability and resistance against chloride ion penetration, which resulted in the enhanced durability of the structures. Therefore, the mixtures are recommended for structures susceptible to chloride and other chemical attacks.
(6) The economic analysis revealed that optimized HPC mixtures incorporating SCMs and fillers significantly enhance cost-effectiveness and mechanical performance, with the optimum quaternary mix achieving the best balance of cost (EUR 75.6/m³) and efficiency (index: 1.047 under hot curing), while the binary and ternary mixes offered tailored improvements for specific conditions.

It is recommended that the effect of water-to-binder ratios above and below 0.3 be investigated in addition to the drying shrinkage and long-term durability studies. Future work could correlate microstructural findings with macro-level performance to better understand the materials’ behavior.

## Figures and Tables

**Table 1:** The chemical and physical properties of the powders used.

Oxide Composition (%)	PC	SF	MQ	FA	LS
SiO_2_	20.2	93.2	99.5	53.20	1.80
Al_2_O_3_	5.49	0.2	0.20	27.3	0.45
Fe_2_O_3_	4.12	0.03	0.03	4.03	0.66
CaO	65.43	0.72	0.01	0.90	54.41
MgO	0.71	0.14	-	0.6	0.41
Na_2_Oeq	0.26	0.07	-	0.95	0.08
SO_3_	2.61	<0.01	-	0.2	0.46
Loss on ignition (%)	1.38	5.4	-	10.02	41.60
Specific gravity	3.14	2.27	2.65	2.45	2.7

**Table 2:** Components (kg/m^3^) of the investigated concrete mixtures.

Mix				Fine Powders					Fine Aggregate		Slump	Temperature	Unit Weight
No.	Type	Fine Powders	Mix ID	PC	SF	MQ	LS	FA	CFS	NFS	mm	°C	kg/m^3^
1	-	PC	CTRL	500	0	0	0	0	251	467	185	25.1	2418
2	Binary	PC-SF	SF10	450	50	0	0	0	245	455	195	25.2	2426
3	Ternary	PC-SF-MQ	SF10-MQ05	425	50	25	0	0	242	449	200	22.3	2430
4			SF10-MQ08	410	50	40	0	0	239	480	192	21.3	2433
5			SF10-MQ10	400	50	50	0	0	239	444	205	19.8	2437
6			SF10-MQ15	375	50	75	0	0	236	438	190	19.8	2441
7		PC-SF-LS	SF10-LS05	425	50	0	25	0	242	449	199	19.4	2408
8			SF10-LS10	400	50	0	50	0	239	444	190	19.5	2401
9			SF10-LS15	375	50	0	75	0	236	438	206	19.2	2394
10		PC-SF-FA	SF10-FA20	350	50	0	0	100	232	432	185	21.7	2435
11	Quaternary	PC-SF-FA-MQ	SF10-FA20-MQ10	300	50	50	0	100	226	420	205	21.5	2421
12			SF10-FA20-MQ08	310	50	40	0	100	227	491	195	21.0	2411
13		PC-SF-FA-LS	SF10-FA20-LS10	300	50	0	50	100	226	420	200	19.7	2431

**Table 3:** Expenses of concrete-making materials.

Material	Approximate Average Cost (EUR/kg)	Source
PC	0.120	[52]
SF	0.410	[53]
MQ	0.230	[53]
LS	0.040	[54]
FA	0.030	[53,54]
CFS	0.025	Local aggregate supplier
NFS	0.020	Local aggregate supplier

**Table 4:** Assessment of the economic efficiency of the analyzed concrete mixtures.

Mix ID	Strength Performance-Based Category	Cost (EUR/m^3^)	Normal Curing		Hot Curing	
			CS (MPa)	Cost-Normalized Index	CS (MPa)	Cost-Normalized Index
CTRL	––––	75.6	84.0	1.111	81.2	1.074
SF10-MQ08	Optimum ternary mix	94.5	89.5	0.947	90.6	0.958
SF10-FA20	Optimum binary mix	79.9	79.0	0.988	81.9	1.025
SF10-FA20-LS10	Optimum quaternary mix	75.6	72.4	0.958	79.1	1.047

## Data Availability

The original contributions presented in this study are included in the article. Further inquiries can be directed to the corresponding author.

## Nomenclature

ASTM	American society for testing and materials
CA	coarse aggregate
CFA	crushed fine aggregate, used in combination with NFA to achieve target gradation
CFS	crushed fine sand
CTRL	control mix without any SCMs or fillers
FA	fly ash
HPC	high-performance concrete
LS	limestone powder
MOE	modulus of elasticity
MQ	micro-quartz
NFA	natural fine aggregate
PC	Portland cement
QM	quartz micro-filler
RCPT	rapid chloride permeability test
SCMs	supplementary cementitious materials
SF	silica fume
SP	superplasticizer
W/B	water-to-binder ratio

## References

[1] Makul N. (2020). Advanced Smart Concrete—A Review of Current Progress, Benefits and Challenges. J. Clean Prod..

[2] Azmee N., Abbas Y.M., Shafiq N., Fares G., Osman M., Iqbal Khan M. (2021). Enhancing the Microstructure and Sustainability of Ultra-High-Performance Concrete Using Ultrafine Calcium Carbonate and High-Volume Fly Ash under Different Curing Regimes. Sustainability.

[3] Abbas Y.M., Khan M.I. (2021). Optimization of Arabian-Shield-Based Natural Pozzolan and Silica Fume for High-Performance Concrete Using Statistical Design of Experiments. Adv. Civ. Eng..

[4] Aïtcin P.C. (2003). The Durability Characteristics of High Performance Concrete: A Review. Cem. Concr. Compos..

[5] Al-Mansour A., Dai Y., Xu C., Yang R., Lu J., Peng Y., Wang J., Lv Q., Zeng Q. (2023). Upcycling Waste Plastics to Fabricate Lightweight, Waterproof, and Carbonation Resistant Cementitious Materials with Polymer-Nano Silica Hybrids. Mater. Today Sustain..

[6] Wang B., Yan L., Fu Q., Kasal B. (2021). A Comprehensive Review on Recycled Aggregate and Recycled Aggregate Concrete. Resour. Conserv. Recycl..

[7] Kaptan K., Cunha S., Aguiar J. (2024). A Review: Construction and Demolition Waste as a Novel Source for CO_2_ Reduction in Portland Cement Production for Concrete. Sustainability.

[8] Belaïd F. (2022). How Does Concrete and Cement Industry Transformation Contribute to Mitigating Climate Change Challenges?. Resour. Conserv. Recycl. Adv..

[9] Hache E., Simoën M., Seck G.S., Bonnet C., Jabberi A., Carcanague S. (2020). The Impact of Future Power Generation on Cement Demand: An International and Regional Assessment Based on Climate Scenarios. Int. Econ..

[10] Müller H.S., Haist M., Vogel M. (2014). Assessment of the Sustainability Potential of Concrete and Concrete Structures Considering Their Environmental Impact, Performance and Lifetime. Constr. Build. Mater..

[11] Alexander M., Beushausen H. (2019). Durability, Service Life Prediction, and Modelling for Reinforced Concrete Structures—Review and Critique. Cem. Concr. Res..

[12] Larsen I.L., Thorstensen R.T. (2020). The Influence of Steel Fibres on Compressive and Tensile Strength of Ultra High Performance Concrete: A Review. Constr. Build Mater.

[13] Farooq F., Nasir Amin M., Khan K., Rehan Sadiq M., Faisal Javed M.F., Aslam F., Alyousef R. (2020). A Comparative Study of Random Forest and Genetic Engineering Programming for the Prediction of Compressive Strength of High Strength Concrete (HSC). Appl. Sci..

[14] de Larrard F., Sedran T. (2002). Mixture-Proportioning of High-Performance Concrete. Cem Concr Res.

[15] Maria da Silva F., Batista L.S., Gachet L.A., Lintz R.C.C. (2022). The Effect of Tire-Rubber Pretreatment on the Physical–Mechanical Properties and Durability of High-Performance Concrete. J. Mater. Civ. Eng..

[16] Khan M.I., Abbas Y.M. (2023). Robust Extreme Gradient Boosting Regression Model for Compressive Strength Prediction of Blast Furnace Slag and Fly Ash Concrete. Mater. Today Commun..

[17] Ismail F.I., Shafiq N., Abbas Y.M., Ateya E.S., Zahid M., Bheel N., Benjeddou O., Abdulkadir I. (2023). The Behavior of Graphene-Nanoplatelets-Based High-Performance Concrete under Ambient Curing. Structures.

[18] Juenger M.C.G., Snellings R., Bernal S.A. (2019). Supplementary Cementitious Materials: New Sources, Characterization, and Performance Insights. Cem. Concr. Res..

[19] Mardani-Aghabaglou A., İnan Sezer G., Ramyar K. (2014). Comparison of Fly Ash, Silica Fume and Metakaolin from Mechanical Properties and Durability Performance of Mortar Mixtures View Point. Constr. Build. Mater..

[20] Alnahhal M.F., Alengaram U.J., Jumaat M.Z., Alsubari B., Alqedra M.A., Mo K.H. (2018). Effect of Aggressive Chemicals on Durability and Microstructure Properties of Concrete Containing Crushed New Concrete Aggregate and Non-Traditional Supplementary Cementitious Materials. Constr. Build. Mater..

[21] Fonseca T.V., dos Anjos M.A.S., Ferreira R.L.S., Branco F.G., Pereira L. (2022). Evaluation of Self-Compacting Concretes Produced with Ternary and Quaternary Blends of Different SCM and Hydrated-Lime. Constr. Build. Mater..

[22] Kumar R., Shafiq N., Kumar A., Jhatial A.A. (2021). Investigating Embodied Carbon, Mechanical Properties, and Durability of High-Performance Concrete Using Ternary and Quaternary Blends of Metakaolin, Nano-Silica, and Fly Ash. Environ. Sci. Pollut. Res..

[23] Sojobi A.O., Awolusi T.F., Aina G.B., Oke O.L., Oladokun M., Oguntayo D.O. (2021). Ternary and Quaternary Blends as Partial Replacement of Cement to Produce Hollow Sandcrete Blocks. Heliyon.

[24] Sabet F.A., Libre N.A., Shekarchi M. (2013). Mechanical and Durability Properties of Self Consolidating High Performance Concrete Incorporating Natural Zeolite, Silica Fume and Fly Ash. Constr. Build. Mater..

[25] Sumesh M., Alengaram U.J., Jumaat M.Z., Mo K.H., Alnahhal M.F. (2017). Incorporation of Nano-Materials in Cement Composite and Geopolymer Based Paste and Mortar—A Review. Constr. Build. Mater..

[26] Kaish A.B.M.A., Odimegwu T.C., Zakaria I., Abood M.M. (2021). Effects of Different Industrial Waste Materials as Partial Replacement of Fine Aggregate on Strength and Microstructure Properties of Concrete. J. Build. Eng..

[27] Larrard F. (1989). de Ultrafine Particles for the Making of Very High Strength Concretes. Cem. Concr. Res..

[28] Kwan A.K.H., Wong H.H.C. (2008). Packing Density of Cementitious Materials: Part 2—Packing and Flow of OPC + PFA + CSF. Mater. Struct..

[29] Qing Y., Zenan Z., Deyu K., Rongshen C. (2007). Influence of Nano-SiO2 Addition on Properties of Hardened Cement Paste as Compared with Silica Fume. Constr. Build. Mater..

[30] Ma B., Li H., Li X., Mei J., Lv Y. (2016). Influence of Nano-TiO2 on Physical and Hydration Characteristics of Fly Ash–Cement Systems. Constr. Build. Mater..

[31] Fu Q., Zhang Z., Zhao X., Xu W., Niu D. (2022). Effect of Nano Calcium Carbonate on Hydration Characteristics and Microstructure of Cement-Based Materials: A Review. J. Build. Eng..

[32] Li H.-W., Wang R., Wei M.-W., Lei N.-Z., Sun H.-X., Fan J.-J. (2022). Mechanical Properties and Hydration Mechanism of High-Volume Ultra-Fine Iron Ore Tailings Cementitious Materials. Constr. Build. Mater..

[33] John V.M., Damineli B.L., Quattrone M., Pileggi R.G. (2018). Fillers in Cementitious Materials—Experience, Recent Advances and Future Potential. Cem. Concr. Res..

[34] Al Martini S., Khartabil A., Sabouni R. (2022). Evaluation of Thermal Conductivity of Sustainable Concrete Having Supplementary Cementitious Materials (SCMs) and Recycled Aggregate (RCA) Using Needle Probe Test. Sustainability.

[35] Du Y., Ge Y. (2021). Multiphase Model for Predicting the Thermal Conductivity of Cement Paste and Its Applications. Materials.

[36] Ramzi S., Hajiloo H. (2022). The Effects of Supplementary Cementitious Materials (SCMs) on the Residual Mechanical Properties of Concrete after Exposure to High Temperatures—Review. Buildings.

[37] (2022). Standard Specification for Portland Cement.

[38] Khan M.I., Abbas Y.M., Fares G. (2023). Enhancing Cementitious Concrete Durability and Mechanical Properties through Sili-ca Fume and Micro-Quartz. Sustainability.

[39] (2006). Standard Test Method for Sieve Analysis of Fine and Coarse Aggregates.

[40] (2021). Standard Test Method for Compressive Strength of Cylindrical Concrete Specimens.

[41] (2022). Standard Test Method for Electrical Indication of Concrete’s Ability to Resist Chloride Ion Penetration.

[42] (2017). Standard Test Method for Temperature of Freshly Mixed Hydraulic-Cement Concrete.

[43] (2017). Standard Test Method for Density (Unit Weight), Yield, and Air Content (Gravimetric) of Concrete.

[44] (2016). Standard Test Method for Flow of Hydraulic Cement Mortar.

[45] Khan M.I., Abbas Y.M., Fares G., Alqahtani F.K. (2023). Flowability and Strength Characteristics of Binary Cementitious Systems Containing Silica Fume, Fly Ash, Metakaolin, and Glass Cullet Powder. Materials.

[46] (2022). Test Method for Static Modulus of Elasticity and Poissons Ratio of Concrete in Compression.

[47] (2021). Testing Concrete—Methods for Analysis of Hardened Concrete.

[48] Akiije I. (2016). Effects of Using 0.5, 0.55 and 0.6 Water Cement Ratio Separately with a Nigerian Grade 42.5 r Portland Cement. Int. J. Sci. Technol. Soc..

[49] Zagar L. Methods of Determination of Pore Structure, Permeability and Diffusion. Proceedings of the RILEM/IUPAC International Symposium on the Pore Structure and Properties of Materials.

[50] Abbas Y.M. (2018). Simplex-Lattice Strength and Permeability Optimization of Concrete Incorporating Silica Fume and Natural Pozzolan. Constr. Build. Mater..

[51] Liao Y., Lv Y., Huang G., Ren S., Wang X.-Y., Guo R., Tian Y., Deng S., Lin R.-S. (2024). Strength and Microstructure Analysis of Subgrade Materials Containing Red Sandstone-Limestone-Cement Composites and Red Sandstone Gravel. Constr. Build. Mater..

[52] Du Y., Korjakins A., Sinka M., Pundienė I. (2024). Lifecycle Assessment and Multi-Parameter Optimization of Lightweight Cement Mortar with Nano Additives. Materials.

[53] Manufacturers, Suppliers, Exporters & Importers from the World’s Largest Online B2B Marketplace. https://www.alibaba.com.

[54] Amazon Publishing. https://www.amazon.com.

